# Turn-taking analysis in patients with schizophrenia: conversational patterns, Self-disorders and the intersubjective dimension

**DOI:** 10.1192/j.eurpsy.2023.1051

**Published:** 2023-07-19

**Authors:** V. Lucarini, F. Giustozzi, N. Fascendini, S. Amorosi, F. Rasmi, F. Magnani, C. Marchesi, F. Cangemi, M. Tonna, M. Grice, M.-O. Krebs

**Affiliations:** 1 Institute of Psychiatry and Neuroscience of Paris (IPNP), INSERM U1266, Université Paris Cité; 2 C’JAAD, Evaluation, Prevention and Therapeutic Innovation Department, GHU Paris Psychiatrie et Neurosciences; 3 Institut de Psychiatrie, CNRS GDR 3557, Paris, France; 4 Psychiatry Unit, University of Parma, Parma, Italy; 5 Institute for Phonetics, University of Cologne, Cologne, Germany

## Abstract

**Introduction:**

Patients with schizophrenia present severe communication difficulties in various linguistic areas. In the last two decades research has invested significant effort in trying to better characterize the linguistic profile of patients with schizophrenia, with the purpose to help and guide diagnosis and treatment. Moreover, speech data could be easily gathered through non-invasive techniques and are therefore seen as particularly promising by clinicians. However, surprisingly very little is known about interactional dialogue management, i.e. turn-taking, in these patients. ‘Schizophrenic autism’, the peculiar intersubjective experience also linked to anomalies in the sense of the self (‘Self-disorders’) presented by these patients, could be at the basis of an unusual turn-taking management.

**Objectives:**

The objective of the present study was to investigate turn-taking patterns of patients with schizophrenia and to explore their possible associations with psychopathological dimensions and subjective experiences.

**Methods:**

We obtained double-channel audio-recordings from interviews with twenty patients with schizophrenia (SCZ) and twenty healthy controls (HC). Participants answered general questions to elicit spontaneous dialogues, to improve the ecological validity of the task. The audio files obtained were then analyzed with Praat, a software widely used in experimental phonetics. We subsequently quantified a set of conversational metrics (participant floor occupation, mutual silence, overlap between speakers, speaking turn and pause duration). Patients also underwent a thorough psychopathological and phenomenological evaluation with the Positive And Negative Syndrome Scale (PANSS), the Examination of Anomalous Self Experience scale (EASE) and the Autism Rating Scale (ARS).

**Results:**

Our results show that the SCZ group displayed a reduced participant floor occupation, an increased mutual silence, and shorter speaking turns as compared to the HC. (Fig. 1, Fig. 2). We found significant associations between conversational features and psychopathology (Fig. 3). Two multivariate linear regressions showed that the participant occupation floor and the average speaking turn duration (dependent variables) were negatively related to the severity of negative symptoms and Self-Disorders. Interestingly, Self-Disorders were the best predictors of conversational engagement.

**Image:**

**Figure:**
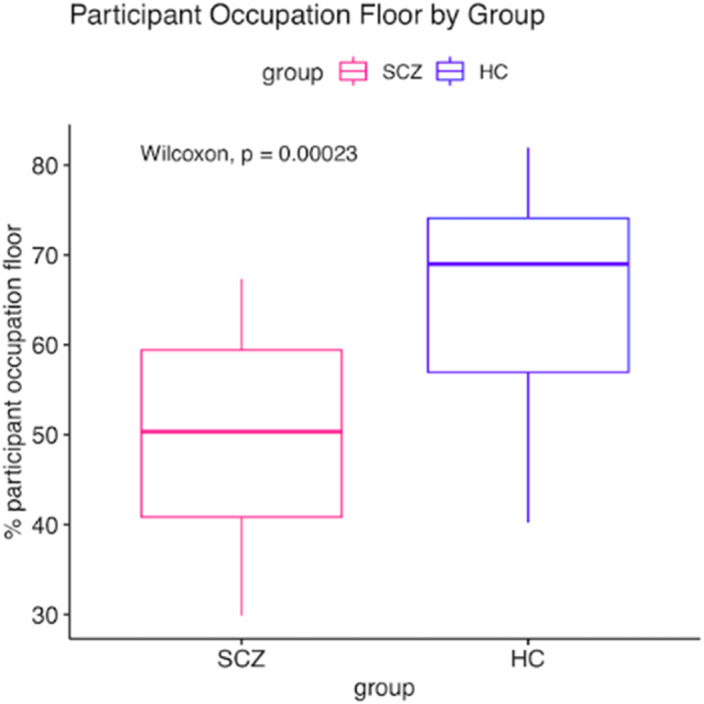

**Image 2:**

**Figure:**
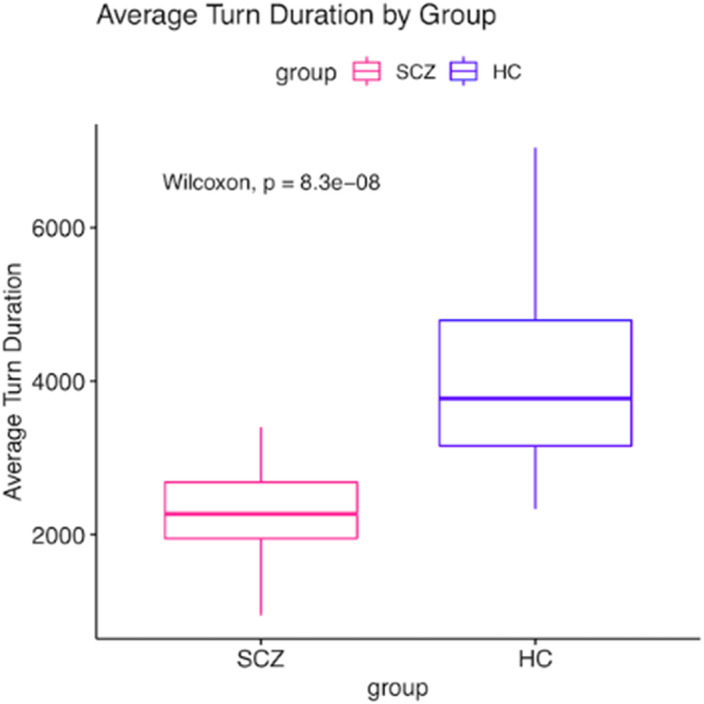

**Image 3:**

**Figure:**
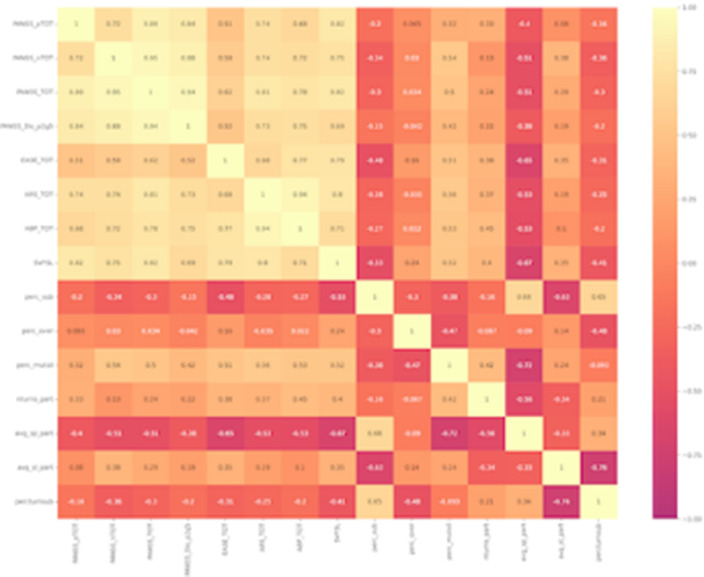

**Conclusions:**

Our results, although preliminary, suggest the existence of peculiar turn-taking patterns in schizophrenia, linked to negative symptoms and disturbances of the subjective experience, particularly in the Self domain. Our results suggest also how the use of experimental linguistic methodology is applicable to clinical settings and underscores the importance of research projects in this field that are strongly interdisciplinary in both design and conduct.

**Disclosure of Interest:**

None Declared

